# Surgical Treatment of Adams Type IV Anterolateral Fracture of the Ulna Coronoid Process

**DOI:** 10.1111/os.13634

**Published:** 2023-01-13

**Authors:** Bo Zhang, Lintao Liu, Junyang Liu, Guangyu Wang, Lei Han, Xu Tian, Jingming Dong

**Affiliations:** ^1^ Department of No. 2 Upper Extremity Traumatology Tianjin Hospital Tianjin China

**Keywords:** Anterolateral fracture, Combined injury, Elbow instability, Surgical treatment, Ulna coronoid process

## Abstract

**Objective:**

Anterolateral coronal fractures are so rare that the mechanism of injury, the type of combined fracture and ligament injury, and the optimal treatment are unknown. To study the outcome of surgical treatments for anterolateral (AL) fracture of the ulna coronoid process (Adams Type IV) and summarize the characteristics of this type of fracture and to guide clinical applications.

**Methods:**

From February 2015 to April 2021, 32 patients were included in the study. All patients had standard radiography with anteroposterior and lateral views, computed tomography, and intraoperative fluoroscopy. All patients were treated surgically. Surgery‐related information, including surgical approach, operation duration, blood loss, and repairing the lateral collateral ligament and the medial collateral ligament integrity, were recorded. The patient's clinical details, such as the final range of motion (ROM), the Broberg–Morrey scores and the visual analogue scale (VAS) at the last follow‐up, were described. The chi‐square test or Fisher's exact test was used for statistical analysis.

**Results:**

We divided patients into two groups according to the anterolateral coronoid fracture morphology. In the intact group, 20 patients with an intact anterolateral coronoid fracture fragment. In the comminuted group, 12 patients with comminuted anterolateral coronoid fracture fragments extended the less sigmoid notch of the ulna. There was no difference in age, sex, total incision length, follow‐up duration, and recovery with rehabilitation among the two groups (all *P*s >0.05). The other follow‐up outcomes, such as elbow ROM (Flexion, Extension, Posterior rotation, Anterior rotation), VAS score, or Broberg–Morrey scores, were not different between the two groups (all *P*s >0.05). Both groups achieved relatively satisfactory clinical outcomes, and the Broberg–Morrey score and index excellence rate reached 84.38%. There is a statistical difference in the history of elbow dislocation (*P* = 0.017), radial head fracture type (*P* = 0.041), operation duration (*P* = 0.014) and blood loss at operation (*P* = 0.029) between the two groups. Cannulated screws, anchors, and sutures were used as point fixation in the coronoid process of the ulna. There was a statistical difference between the two groups in the choice of internal fixation (*P* = 0.020).

**Conclusions:**

For anterolateral ulnar coronoid fractures with different degrees of comminution, effective and reliable surgical treatment can achieve better results and fewer complications.

## Graphical abstract

We subdivided the anterolateral coronoid fragment intact as Type IV(AL)a, and comminuted anterolateral coronoid fragment involving the lesser sigmoid notch of ulna is defined as Type IV(AL)b.

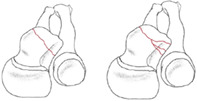

## Introduction

Ulna coronoid process fractures are caused by shear or rotational force to the elbow when the elbow is under excessive pressure and is usually the result of a dislocation of the elbow joint. Coronoid process fractures are relatively rare and occur in about 2%–15% of patients with elbow dislocation.^1^ Regan and Morrey originally described three types of coronoid process fractures.^2^ Type I is described as avulsion of the tip of the coronoid process, Type II fractures involve less than 50% of the coronoid process, and Type III fractures involve more than 50% of the coronoid process. O'Driscoll *et al*.^3^ then modified the Regan and Morrey classification of the ulna coronoid process fracture to Type I transverse fracture of the tip of the coronoid process, Type II anterior medial fracture of the coronoid process, and Type III basal fracture of the coronoid process. We have clinically observed an anterolateral coronoid process fracture with an oblique fracture line extending to the lesser sigmoid notch of the ulna, which is not described by the above two classification systems. Butler *et al*.^4^ first reported a case of an anterolateral fracture of the ulna coronoid process and treated it conservatively. Adams *et al*.^5^ used CT scans to describe the morphology of coronoid process fractures; they reported that only 7% of the studied population had anterolateral coronoid process fractures, defined as Type IV(AL), but the study did not involve clinical treatment.

The coronoid process is one of the major stabilizing structures of the humeroulnar joint. The coronoid process and the radial head support the elbow joint; the radial head prevents valgus instability, and the coronoid process prevents varus instability. Isolated coronoid anterolateral fractures are rare. They usually occur with injuries to other structures around the elbow, and the coronoid process is very important for maintaining elbow joint stability.^6^ Ligament damage associated with coronoid process fractures may play a more important role in elbow instability than the fracture itself. Some authors recommend surgical fixation of most coronoid process fractures.^7^

Surgical treatment of anterolateral coronoid process fractures has been rarely reported, and appropriate surgical treatment methods, including surgical approach and internal fixation methods, are still unknown. Since this type of fracture is very rare, this study was a retrospective study on the surgical treatment of Adams Type IV anterolateral fracture of the ulna coronoid process, a comparative study of the clinical characteristics of fractures with different degrees of comminuted, and an analysis of the injury characteristics patterns and proposed a treatment algorithm based on fracture morphology, ligament injury pattern etc. Therefore, the purposes of this study can be described as follows: (i) to investigate injury mechanism and clinical characterization of anterolateral coronoid process fracture; (ii) to evaluate the efficacy of different surgical treatments for this type of fracture; and (iii) to analyze the different surgical approaches and internal fixation for comminuted coronoid fractures.

## Materials and Methods

### Inclusion and Exclusion Criteria

Inclusion criteria include: (i) older than 18 years old; (ii) clinical examination and full imaging study at administration (three‐dimensional reconstruction CT scans) confirmed anterolateral coronoid process fracture; (iii) acute fracture within 2 weeks of injury; and (iv) patients with at least clinical follow‐up for more than 12 months. Complete follow‐up outcomes, including range of motion (ROM) and elbow function scores.

Exclusion criteria are: (i) pathological fractures; and (ii) multiple fractures in other parts of the ipsilateral limb.

### General Information

This research was reviewed and approved by the Tianjin Hospital Medical Ethics Committee (2022 Medical Ethics Review 089). It constitutes a retrospective study on the surgical treatment of 376 patients with ulna coronoid process fractures who were admitted to the emergency department between February 2015 and April 2021.

### Imaging Evaluation

All patients underwent standard X‐ray (frontal and lateral), CT scans, and intraoperative fluoroscopy. Three‐dimensional reconstruction CT scans diagnosed the anterolateral fracture of the coronoid process as follows: (i) The distal part of the humerus was subtracted, and observed the coronoid from top to bottom; and (ii) CT scans showing an anterolateral oblique fracture line from the anteromedial to the posterolateral of the coronoid process involve the less sigmoid notch of the ulna.^5^ CT scans were observed by three surgeons, with consensus by at least two of them.

### Patient Classification

**Fig. 1:**
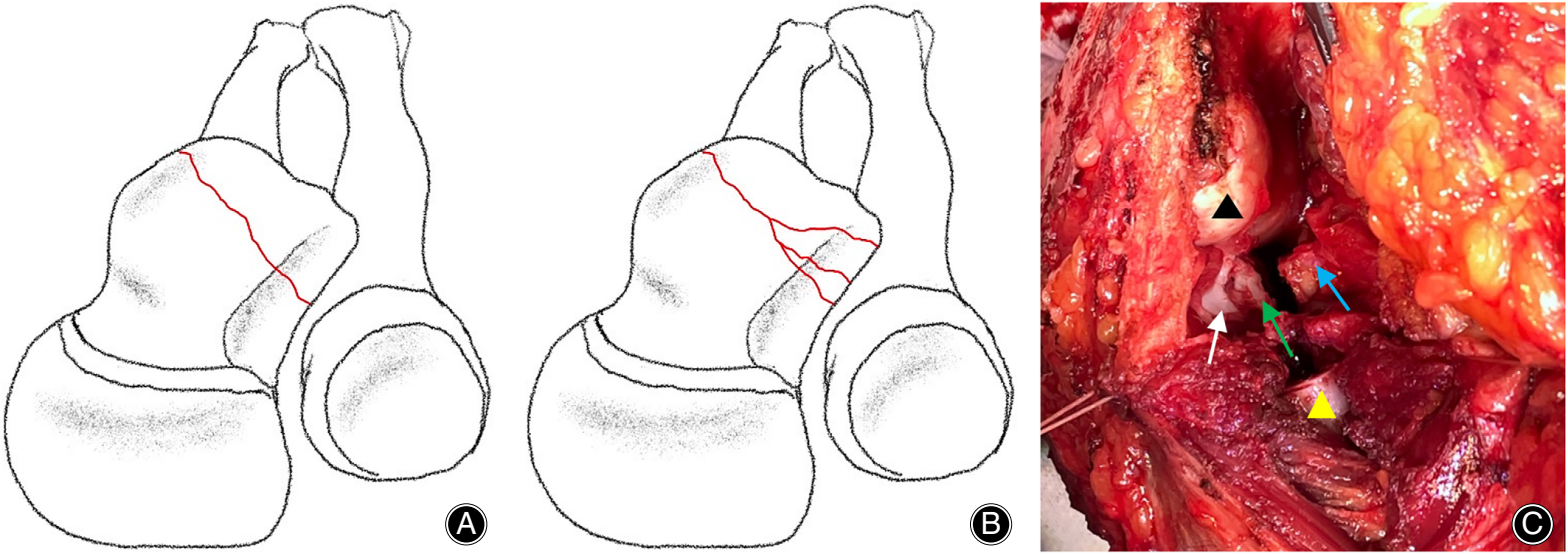
(A) The anterolateral coronoid fragment intact is defined as Type IV(AL)a. (B) The anterolateral coronoid fragment comminuted especially the less sigmoid notch of the ulna comminuted is defined as Type IV(AL)b. (C) Removal of the radial head to expose the Type IV(AL)b fracture during radial head replacement through the Kocher approach. The anterolateral coronoid fracture involved the tip of coronoid (blue arrow), comminuted less sigmoid notch of ulna (green arrow), base of the coronoid (white arrow) (black triangle: distal humerus; yellow triangle: radial neck).

Adams classification^5^ has divided the coronoid process fracture into five types. Type I: tip fracture. Type II: mid‐transverse fracture. Type III: fracture of the coronoid base. Type IV(AL): anterolateral fracture. Type IV(AM): anteromedial fracture. We subdivided the anterolateral coronoid fragment intact as Type IV(AL)a, and the comminuted anterolateral coronoid fragment involving the lesser sigmoid notch of the ulna is defined as Type IV(AL)b (Fig. 1). The Mason classification method divides radial head fractures into three types. Type 1 is a nondisplaced fracture. Type 2 is a fracture with partial displacement. Type 3 is a displaced fracture involving the entire radial head.^8^ The Mayo classification method classifies ulna olecranon fractures as follows: Type I, nondisplaced fractures, treated non‐operatively; Type II, displaced, stable fractures that require operative fixation; and Type III, displaced, unstable fractures.^9^

The presence of ulnohumeral dislocation, lateral collateral ligament (LCL) and medial collateral ligament (MCL) damage, and other information was also recorded.

### Surgical Treatment

After patients checked into the emergency room, the doctor examined their ipsilateral shoulder, elbow, and wrist to see if the elbow joints were dislocated. An X‐ray image of the positive side of the elbow joint was taken for each patient. For patients with combined humeroulnar joint dislocation, the closed reduction was performed under anesthesia, which is more beneficial to the patient. After the reduction, the upper limb was attached to an elbow brace. If the plaster was used for fixation, attention was paid to the skin's surface tension across the elbow joint.

The surgical treatment of these fractures has been carried out by one of the three participating surgeons specializing in upper extremity trauma with more than 10 years of experience. All 32 patients received surgical treatment. All patients are in a supine position when being treated.

#### Surgical Approach

The surgical approach is selected based on the combined fracture. The Boyd approach^10^ is used to observe and fix the coronoid process fracture through the olecranon fracture for patients with combined olecranon fractures. For patients with radial head fractures or other radial column fractures, the Kocher approach is used to reduce and fix the coronoid process, then fix or reconstruct the radial head, and finally explore and repair the LCL. If the reduction and repair of coronoid fractures cannot be performed using the Boyd or Kocher approach, the anteromedial elbow approach can completely expose the coronoid process. There was no anterior dislocation or loosening of the ulnar nerve *in situ*. For patients without other fractures, the Kocher approach is preferred to explore and repair the LCL.

#### Internal Fixation Options

The choice of internal fixation is based on the degree of comminution of the coronoid fracture. When the fragment is intact and small, 2.5 or 3.0 mm cannulated screws can fix the coronoid process. For extremely small, crushed fragment fractures, 3.0‐mm anchors or Ethibond suture lasso to the joint capsule can be used to fix the coronoid process. When the fragment is large and crushed, the locking plate (Biomet F3 microplate) can fix the coronoid for better reduction, fixation and support.

#### Ligament Repair

The ligament repair is determined according to the stability of the elbow joint during the operation. The Kocher approach can also explore and repair the LCL and resolve other fractures of the lateral column of the elbow. After immobilization, an apprehension test was performed under the C‐arm to monitor elbow stability. If the elbow joint is stable, MCL may not be repaired. A medial approach is required to explore and repair the MCL if the elbow remains unstable. When using the anteromedial approach, repair the MCL if necessary. The elbow varus stress test was performed under the C‐arm after immobilization. If the widening of the humeroradial joint space indicates damage to the LCL, the Kocher approach is used to explore and repair the LCL.

## Postoperative Care

After keeping the elbow at 90‐degree flexion for 3 days after surgery, active and passive flexion and extension activities are allowed while using a mobile brace for protection. The elbow extension should be no more than 30°, and the forearm should be kept in a neutral position. After 3 weeks, active flexion and extension exercises continued with the flexion and extension angle gradually increasing, and at the same time, starting with the forearm rotational function exercises.

### Postoperative Follow‐Up and Outcomes Measurement

#### Range of Motion

Clinically, standard procedures measure the ROM, including flexion, extension, pronation and supination. The normal range of extension and flexion is from approximately 0° to 145°–150°. Normal pronation and supination are approximately 80° and 90°, respectively.

#### Broberg–Morrey Scores and Indexes

The Broberg–Morrey rating system^11^ is a 100‐point system, which consists of four parts: exercise (40 points), strength (20 points), stability (5 points), and pain level (35 points). The scores are as follows: 95–100 points indicate excellent; 80–94 points, good; 60–79 points, fair; ≤60 points, poor. If the score is rated as good or excellent, the result can be considered satisfactory, and if the score is fair or poor, the result is considered unsatisfactory.

#### Pain Level Assessment by Visual Analogue Scale

A 10‐cm long ruler is marked with 10 graduation points with “0” points at one end and “10” points at the other end. 0 points indicate no pain, and 10 points indicate the most severe pain that is unbearable. During the test, patients placed the cursor on the area that best represented the degree of pain at that time, and the doctor scored based on the position marked by the patient. 0 is no pain, 1–3 is mild, 4–6 is moderate, and 7–10 is severe.

#### Valgus and Varus Stability

We tested the valgus and varus stability at maximum extension and 30° flexion. The pivot shift test evaluates the posterolateral rotation's stability and is scored using four levels: normal, mild, moderate, and severely unstable.^12^

#### Complications Related to Surgery: Infection, Non‐healing Fractures, Ulnar Nerve Damage

All patients have regularly observed wounds after the operation, whether there was purulent exudation. All patients were examined using anteroposterior (AP) and lateral view X‐ray scans of the injured elbow during follow‐up. The bridge bone formation shown in the AP and lateral X‐rays indicated healing of the fracture. During follow‐up, patients were checked for hand deformities, ulnar sensory loss, or restricted ring motion‐ and little finger. Ultrasound and electromyography (EMG) confirmed the presence or absence of ulnar nerve injury.

### Statistical Analysis

SPSS version 26.0 software (IBM Corporation, Chicago, IL, USA) was used for the statistical analysis of the data. All measurement data are expressed as the mean ± standard deviation. All quantitative data are calculated both as numbers and percentages. The chi‐square test, *t*‐test or Fisher's exact test were used for statistical analysis. A value of *P* < 0.05 indicates that the difference between research groups is statistically significant.

## Results

### General Information

From February 2015 to April 2021, 376 patients with ulna coronoid process fractures were admitted for surgical treatment, of which 34 had anterolateral coronoid process fractures, with an incidence rate of 9.04%. There were two missed follow‐ups, with a missed visit rate of 5.88%, and 32 patients were finally included. There were 17 cases of men and 15 cases of women, with an average age of 46.5 years (the age range: 18–76 years). The average follow‐up time was 36.34 months (range: 12–86 months), with 28 cases of falls from a standing height, one case of high‐altitude fall injuries, and three cases of car accidents.

For combined injuries, there were 28 cases combined with other fractures of the ipsilateral elbow joint (87.50%), including 11 cases of ulna olecranon fractures (34.38%), 22 cases of radial head fractures (68.75%), 11 cases of lateral humerus condyle avulsion fracture (34.38%), two cases of medial humerus condyle avulsion fracture (6.25%), and one case of capitellum fracture (3.13%). There were 28 cases (87.50%) of damage repair of the LCL of the ipsilateral elbow joint and 10 cases (31.25%) of damage repair of the MCL. There was one case of combined injury with ulnar nerve injury (3.13%), one case of concomitant injury with pelvic fracture (3.13%), 18 cases of combined injury with humeroulnar joint dislocation (56.25%), and two cases of combined injury with upper ulnar radial joint dislocation (6.25%). The injury mechanisms consisted of 13 cases (40.63%) of unstable posterolateral rotation, eight cases (25.00%) of ulna olecranon fractures and dislocations and two cases (18.18%) of Monteggia fracture.

**Fig. 2:**
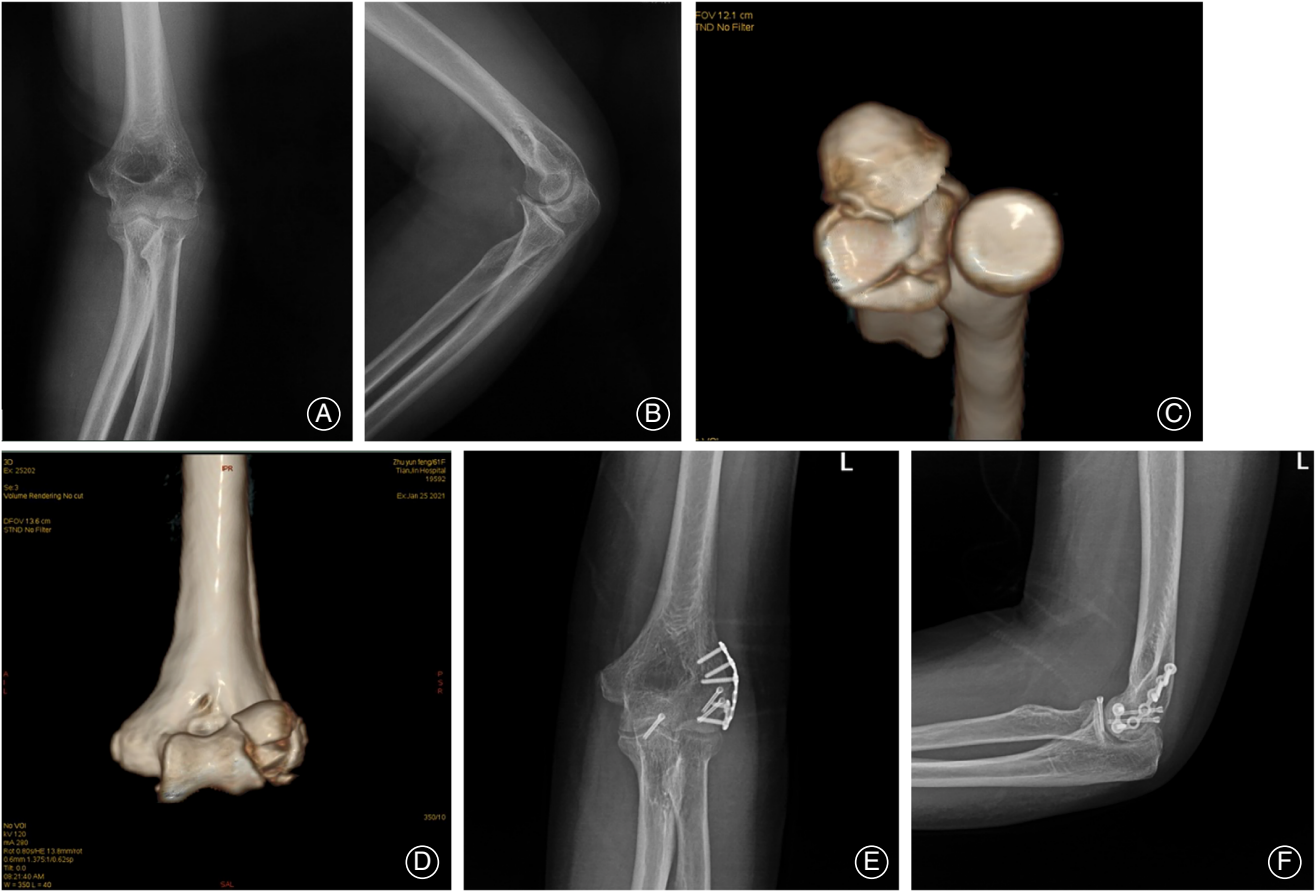
Patient No. 16, the only case combined with capitellum fracture in this study. A 61‐year‐old woman fell from the standing height and injured her left non‐dominant elbow. (A, B) Initial anteroposterior and the lateral radiograph showed a suspected fracture of the coronoid process of the ulna. (C, D) A computed tomography image showed an Adams Type IV(AL)a fracture of the coronoid process and the capitellum fracture. (E, F) The anteroposterior and lateral radiographs that were obtained following the operative treatment.

**Fig. 3:**
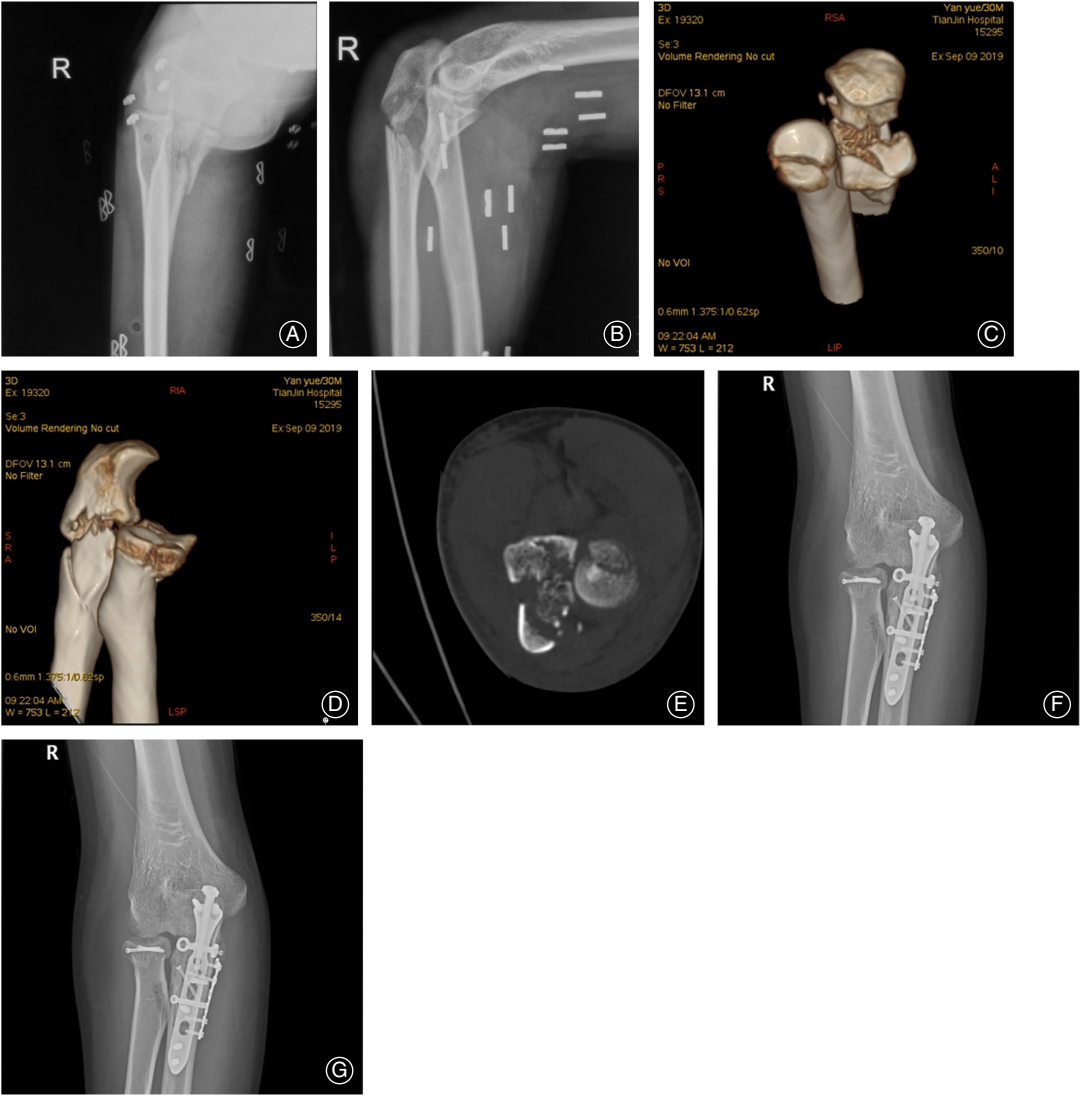
Patient No. 27. A 30‐year‐old man fell from the standing height and injured his right dominant elbow. (A, B) Initial anteroposterior and the lateral radiograph showed proximal ulna fracture and radial head fracture. Standard anteroposterior and the lateral radiographs cannot be obtained due to severe pain in the patient. (C, D, E) A computed tomography image showed that an Adams Type IV(AL)b fracture of the coronoid process. (F, G) The anteroposterior and lateral radiographs that were obtained following the operative treatment.

We divided patients into two groups according to the anterolateral coronoid fracture morphology. In the intact group, 20 patients with intact anterolateral coronoid fracture fragments (Fig. 2); in the comminuted group, 12 patients with comminuted anterolateral coronoid fracture fragments which extended the less sigmoid notch of the ulna (Fig. 3). There is no difference based on age (*P* = 0.586), sex (*P* = 0.234), total incision length (*P* = 0.314), follow‐up duration (*P* = 0.092), and modalities of rehabilitation (*P* = 0.926) among the two groups (Table 1). The other outcomes such as elbow ROM (Flexion *P* = 0.888; Extension *P* = 0.894; Posterior rotation *P* = 0.333; Anterior rotation *P* = 0.638), visual analogue scale (VAS) score (*P* = 0.337), or Broberg–Morrey scores (*P* = 0.422) are not different among the groups (Table 2). Both groups achieved relatively satisfactory clinical outcomes, and the Broberg–Morrey score and index excellence rate reached 84.38%. There is a statistical difference in combined with elbow dislocation (*P* = 0.017), radial head fracture type (*P* = 0.041), operation duration (*P* = 0.014) and blood loss at operation (*P* = 0.029) between the two groups (Table 3). Cannulated screws, anchors, and sutures were used as point fixation in the coronoid process of the ulna. There was a statistical difference between the two groups in the choice of point and plate fixation (*P* = 0.020).

**TABLE 1:** Baseline data

Variable	Level	Intact group	Comminuted group	*t*/*x* ^2^	*P*
*N*		(20)	(12)		
Age (mean [SD])		47.85 ± 16.99	44.25 ± 19.38	0.551	0.586
Sex (%)	Male	9 (45.0)	8 (66.7)	1.414	0.234
	Female	11 (55.0)	4 (33.3)		
Combined with elbow dislocation (%)	No	12 (60.0)	2 (16.7)	5.723	0.017^a^
	Yes	8 (40.0)	10 (83.3)		
Radial head classification by Mason (%)	II	4 (30.8)	0 (0.0)		0.041^b^
	III	3 (23.1)	0 (0.0)		
	IV	6 (46.2)	9 (100.0)		
Olecranon classification by Mayo (%)	IIB	1 (33.3)	0 (0.0)		0.161^b^
	IIIA	2 (66.7)	4 (57.1)		
	IIIB	0 (0.0)	3 (42.9)		

^a^ Chi‐square test.

^b^ Fisher's exact probability test.

**TABLE 2:** Surgical treatment information

Variable	Level	Intact group	Comminuted group	*t*/*x* ^2^	*P*
*N*		(20)	(12)		
Coronoid internal fixation (%)	Point	15 (75.0)	4 (33.3)	5.398	0.020^a^
	Plate	5 (25.0)	8 (66.7)		
LCL repair (%)	No	2 (10.0)	2 (16.7)	0.305	0.581^a^
	Yes	18 (90.0)	10 (83.3)		
MCL repair (%)	No	14 (70.0)	8 (66.7)	0.039	0.844^a^
	Yes	6 (30.0)	4 (33.3)		
Operation duration, h (mean ± SD)		1.90 ± 0.53	2.42 ± 0.56	−2.625	0.014
Blood loss at operation, ml (mean ± SD)		75.00 ± 41.36	120.83 ± 72.17	−2.294	0.029
Total incision length, cm (mean ± SD)		18.35 ± 8.87	21.58 ± 8.23	−1.025	0.314
Follow up duration, mon (mean ± SD)		41.75 ± 26.71	27.33 ± 12.94	1.743	0.092
Rehabilitation after operation (%)	No	12 (60.0)	7 (58.3)	0.009	0.926^a^
	Yes	8 (40.0)	5 (41.7)		

Abbreviations: LCL, lateral collateral ligament; MCL, medial collateral ligament.

^a^ Chi‐square test.

**TABLE 3:** Description of primary indicators (mean ± SD)

Variable	Intact group	Comminuted group	*t*	*P*
	(20)	(12)		
Flexion	145.00 ± 13.95	144.17 ± 19.29	0.142	0.888
Extension	8.50 ± 11.48	7.92 ± 12.70	0.134	0.894
Pronation	82.50 ± 13.72	77.50 ± 14.22	0.985	0.333
Supination	80.00 ± 5.62	79.17 ± 2.89	0.475	0.638
VAS score	0.95 ± 1.15	0.50 ± 1.45	0.975	0.337
Broberg–Morrey score	87.55 ± 12.00	91.08 ± 11.68	−0.814	0.422

Abbreviation: VAS, visual analogue scale.

### Complications

At the follow‐up, no other complications (e.g., infection, non‐healing fractures) were detected in any patients. There were two cases of ulnar nerve injury in the comminuted group. Patient No. 5 experienced loss of sensation on the ulnar side of the ring‐ and little fingers after surgery, which was diagnosed and confirmed as ulnar nerve injury by EMG examination. Patient No. 21 had an occasional loss of sensation in his little finger 6 months after the operation, which was related to the intensity of rehabilitation exercise. Two patients were given methylcobalamin treatment to improve nerve nutrition, and their symptoms improved after ulnar nerve release. Intraoperatively, scarring surrounding the ulnar nerve was found to cause nerve compression.

## Discussion

In our study, there was a statistical difference in internal fixation between the two groups, with plate fixation being used more in comminuted group. Regarding fracture severity, the comminuted group was more comminuted than the intact group, but the final elbow ROM, VAS score, and Broberg–Morrey scores showed no statistical differences. This may be related to the fact that plate fixation provides more effective fixation and support and enables early functional exercise. Comminuted fractures of the ulna's radial notch may cause forearm rotation impairment. Among the 32 patients, patient No. 24, with the worst rotational function (rotation range 110°, post‐rotation 40°, pre‐rotation 70°), was in the comminuted group. However, there was no statistical difference in rotational function between the two groups in this study. Therefore, the effect of comminuted fracture of the ulna radial notch on rotational function needs to be verified by a larger sample size study.

### Incidence and Mechanism

Ulna coronoid anterolateral fractures are rare. They are often combined with other injuries. The incidence of anterolateral coronoid process fractures in this study is 9.04%. Although this value is higher than the 7% incidence reported by Adams *et al.*,^5^ it is still a relatively rare type of coronoid process fracture. In this study, more than 50% of the patients had combined humerus‐ulnar joint dislocation (56.25%). This particular type of injury mechanism is worthy of further discussion. This injury mechanism involves elbow dislocation, fracture type, and intraoperative exploration of the ligament. It is known that the common elbow joint injury mechanism is used to classify patients. Most of the 11 patients with olecranon fracture also suffered from the mechanism of trans ulnar olecranon fracture with dislocation (72.73%). Two patients with Monteggia injuries (18.18%), consistent with Liu *et al*.'s^13^ description, had comminuted epiphysis fractures. Most of the injury mechanisms of the 21 patients with non‐ulnar olecranon fractures were unstable posterolateral rotation^14^ (61.9%).

### Surgical Indications

For isolated fractures with little displacement of the anterolateral coronoid process, no ligament injury, and stable elbow joint, conservative treatment can be tried, and successful cases have been reported.^4^ In this study, there were seven cases of isolated anterolateral fractures. However, the fear test after injury or preoperative anesthesia showed that the elbow joint was unstable, or the elbow joint varus test showed that the humeroradial joint space was widened performed on the C‐arm, so surgical treatment was selected. For patients with other fractures, the injury of the lateral column or the posterior column of the elbow joint will affect the stability of the elbow joint. Ensuring stability is the first principle in dealing with elbow joint injuries. Both two groups achieved relatively satisfactory clinical outcomes, and the Broberg–Morrey score and index good and excellence rate reached 84.38%. Both can obtain good elbow joint mobility, VAS score, and function score and improve the quality of life.

### Choice of Approach and Internal Fixation

According to the three‐column theory of the elbow joint,^15^ most of the anterolateral coronoid fractures in this study involved lateral column fractures (84.38%), including the radial head and the lateral condyle of the humerus. It is usual to stabilize the lateral side first, to downgrade the injury, then reassess and stabilize the medial side of the elbow that remains unstable.^16^ Unstable elbow joints with coronoid process tip fractures can be fixed directly through the lateral Kocher approach to expose the lateral epicondyle and main extensor tendon. Torn extensor tendons usually accompany severe triple injuries. This method can be used to expose the LCL and the radial head. After exposing the fracture of the radial head, we can reduce, fix or replace it. When the radial head is crushed and needs to be removed, we may be able to easily visualize the anterolateral fracture of the coronoid process. When only part of the radial head is broken, the visualization of the coronoid process is quite difficult. Sometimes it is necessary to cut part of the anterior joint capsule, then use a 3.0‐mm anchor to repair the joint capsule. Deutch *et al*.^17^ checked the stability of the elbow when subjected to rotational stress. When the coronoid process is broken, and the radial head is removed, the joint will be semi‐dislocated regardless of the state of the collateral ligament. Even without a radial head, LCL reconstruction can prevent severe relaxation. The replacement of the radial head and the reconstruction of the LCL lead to greater stability. The LCL is the main stabilizer for external rotation. The lateral approach is used to fix the ulna coronoid process fracture, and the LCL can be explored simultaneously. If the ulnar pulley joint can extend fully without dislocation under gravity after repairing the coronoid process, the radial head, and the LCL, there is usually no need to explore and repair the MCL. Even in elbow varus posteromedial rotatory instability, where the medial column is the main injury, satisfactory functional outcomes can be yielded with non‐repair of the LUCL as long as the stable elbow joint is performed during the operation.^18^ After the anterior medial approach is used to fix the coronoid process fracture, an intraoperative valgus test is required: using the C‐arm to monitor the widening gap between the lower humerus‐radial joint. If the valgus test is positive, the lateral approach must be used to repair the LCL. This study's combined LCL damage rate was as high as 87.50%. Because of the above facts, we think that the Kocher approach makes it easier to explore the damage and repair of the LCL. However, the Kocher approach cannot use a plate to fix the coronoid process for stronger and more stable support. We need an anteromedial approach.

Larger fractures in the base of the coronoid process can be fixed with screws. The screws are fixed at the plate behind the olecranon. Using the anterior cruciate ligament (ACL) as a drill guide simplifies this process so cannulated screws can be inserted from behind the ulna toward the front. For finely broken coronoid fractures, sutures can be used to fix the pointed fractures, and multiple plates can be used to support them. Ochtman and Ring^19^ described the use of posterior and medial double plates to fix the proximal ulna fracture. Disadvantages of using the medial plate include ulnar nerve damage, extensive medial muscle stripping and non‐healing fractures, and the possibility of screws penetrating the humerus‐ulnar joint and the upper ulnar radial joint.^19^ Therefore, when we use the anterior medial, and Boyd approaches, we need to pay special attention to protecting the ulnar nerve.

### Strengths and Limitations

The anterolateral fracture of the ulna coronoid process is a newly recognized rare sub‐type of the coronoid fracture. This subtype is usually a part of the complex elbow fracture. The study's strength relies not only upon the pre‐operation CT study and the detailed surgical records but also on the evaluation of full follow‐up post‐operation with objective elbow function.

However, this study is a retrospective analysis, and due to the extremely low clinical incidence of anterolateral ulna coronoid fractures, the study sample size is small. Since other treatment methods are not included, and the intensity of rehabilitation exercises is inconsistent, there are inevitable systematic and selectivity errors. The sample size needs to be further expanded to confirm our results. The injury mechanisms can also be determined by finite element analysis. In addition, the Adams classification does not describe the radial notch of the ulna fracture that does not involve the tip of the coronoid process. Further classification studies should be carried out according to the size and location of the anterolateral bone fragment to analyze the injury mechanism and treatment strategies. Despite the small number of cases included in the present treatment analysis, the new information obtained from this study can still provide a valuable guide for practical clinical applications.

### Conclusion

Ulna coronoid anterolateral fractures isolated are rare. They are often combined with other injuries to the radial side of the elbow joint. Clinically, it is necessary to analyze the injury mechanism and fracture type according to the size of the fracture fragment, the location of the bone fragment, the type of combined fractures and ligament injury, the history of dislocation of the elbow joint, etc., and choose the best treatment plan according to the patient's situation. For anterolateral ulnar coronoid fractures with different degrees of comminution, effective and reliable surgical treatment can achieve better results and fewer complications.

## Author Contributions

All authors agree with the manuscript. Bo Zhang and Lintao Liu acquired and wrote the manuscript, Junyang Liu analyzed data, Xu Tian and Jingming Dong performed the surgery. Lei Han and Guangyu Wang collected data.

## Ethics Statement

This research was reviewed and approved by the Tianjin Hospital Medical Ethics Committee (2022 Medical Ethics Review 089).

## Funding Information

This study was Funded by Tianjin Key Medical Discipline(Specialty) Construction Project (TJYXZDXK‐026A).

## Conflict Of Interest

The authors declare that they have no conflict of interest related to the publication of this manuscript.
